# Seeing Through the Mimicry of *Papilio bootes* by Combining Computer‐Aided and Human Eyesight Morphological Comparisons

**DOI:** 10.1002/ece3.72369

**Published:** 2025-10-30

**Authors:** Yuan‐Rui‐Xue Xie, Zhi‐Xing Ding, Adam M. Cotton, Zhen‐Bang Xu, Yue Pan, Yi‐Ting Lin, Shao‐Ji Hu

**Affiliations:** ^1^ Institute of International Rivers and Eco‐Security Yunnan University Kunming China; ^2^ Yunnan Key Laboratory of International Rivers and Transboundary Eco‐Security Yunnan University Kunming China; ^3^ Asian International River Center Yunnan University Kunming China; ^4^ College of Ecology and Environment Yunnan University Kunming China; ^5^ School of Information Science and Engineering Yunnan University Kunming China; ^6^ Independent Scholar Chiang Mai Thailand

**Keywords:** Batesian mimicry, *Byasa*, deep learning, human visual assessment, *Menelaides*

## Abstract

The Tailed Redbreast *Papilio bootes* exhibits a tendency for specific mimicry of sympatric *Byasa* species across its distribution range, but this phenomenon has not yet been quantitatively analysed. To address this intriguing example of Batesian mimicry, the present study focused on three provinces (Yunnan, Sichuan, and Shaanxi) in West China, which have high taxonomic diversity of both *P. bootes* and *Byasa*. We combined computer‐aided and human eyesight morphological comparisons to reveal the visual similarity between five subspecies of *P. bootes* and 13 species of *Byasa*. Our findings demonstrate that the subspecies *mindoni* and *parcesquamata* of *P. bootes* in the western part of its range primarily mimic *Byasa* species with four hindwing white spots ( *B. latreillei* , *B. polla* and *B. genestieri*), while the black subspecies *nigricauda* and *dealbatus* in the northeastern part of the range specifically mimic *Byasa* species without hindwing white spots (*B. impediens*, *B. plutonius* and black *B. polyeuctes*). Between these two extremes, the subspecies *rubicundus* and the spotted *nigricauda* mimic several different *Byasa* species, with the variable *B. polyeuctes* acting as the morphological bridge between them. Considering the phylogenetic history of *Byasa* and *P. bootes*, the authors propose that the four‐spotted trait in *P. bootes* could be ancestral, while the spotless trait may have resulted from subsequent selection. The widespread and morphologically variable *B. polyeuctes* may enhance its own fitness by potential Müllerian mimicry and also serve as a bridge to mingle *P. bootes* and various *Byasa* species through Batesian mimicry in the transition area between four‐spotted and spotless *Byasa* species.

## Graphical abstract

The study investigates Batesian mimicry between *Papilio bootes* and *Byasa* species in West China, revealing that different subspecies of *P. bootes* mimic *Byasa* species with varying hindwing spot patterns. Subspecies in the western range mimic four‐spotted *Byasa* species, while northeastern subspecies mimic spotless ones, with *B. polyeuctes* acting as a transitional bridge. The findings suggest the four‐spotted trait in *P. bootes* may be ancestral, with spotless traits evolving later, and *B. polyeuctes* potentially enhancing fitness through both Batesian and Müllerian mimicry.

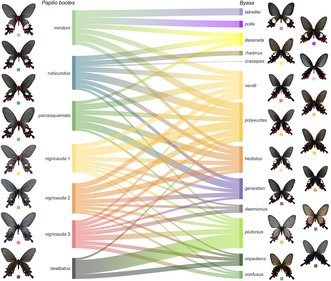

## 1 Introduction

Batesian mimicry refers to a phenomenon that nontoxic or palatable species in nature evolved to have a resemblance in shape, colour and texture to the toxic or unpalatable species to avoid predation (Mallet and Gilbert 1995; Outomuro et al. 2016; Ruxton et al. 2019). Batesian mimicry is an example of adaptation under natural selection and is an important aspect in biological research (Bonner 1988; Prudic et al. 2007; Futahashi and Fujiwara 2008). This phenomenon is common in nature but much more noticeable in butterflies (Kristensen et al. 2007), which has long been a focus of evolutionary biology research (Anderson and de Jager 2020).

Visual features are crucial in Batesian mimicry, particularly the colour patterns of butterfly wings. These patterns, including complex elements like stripes and spots with diverse shapes and colours resulting from coevolution (Turner 1984; Nishida and Fukami 1989; Nijhout 2001; Kunte 2009; Kronforst and Papa 2015), facilitate visual identification by predators. This visual signalling both prevents prey from being taken and serves to confuse or warn predators (Chai 1996; Basu et al. 2023). During the evolution of butterfly patterns, the same set of individual pattern elements is arranged in new ways to produce species‐specific patterns, including adaptations such as mimicry and camouflage (Nijhout 2001).

Predators perceive visual features to evaluate the palatability of the preys. Unpalatable butterflies ward off predators by displaying distinctive wing patterns, such as the banded markings in the genus *Heliconius* (Nymphalidae: Heliconiinae) as a means of announcing toxicity (Nijhout 1991). The white‐spotted mimicry of the unpalatable *Pachliopta aristolochiae* to avoid predation by female *Papilio polytes* in the family Papilionidae has also been validated by a series of studies (Clarke and Sheppard 1972; Mallet 2008; Kunte 2009; Song and Liang 2009; Nishikawa et al. 2013). Brower (1958) studied the Batesian mimicry phenomenon between *Limenitis archippus* and *Danaus plexippus* and found that neither *D. plexippus* nor *L. archippus* was attacked or even approached in any experiment, indicating that the resemblance in colour pattern is linked to its unpalatability.

Among the numerous cases of Batesian mimicry in butterflies, a particularly interesting example is the mimicry between different subspecies of the Tailed Redbreast *Papilio bootes* and various *Byasa* species. Throughout its distribution range, *P. bootes* exhibits high variability in hindwing markings that can be divided into eight subspecies, namely ssp. *bootes* in Meghalaya, N.E. India; ssp. *mixta* in Nagaland, N.E. India; ssp. *mindoni* in Kachin State, N. Myanmar and the Dulongjiang‐Irrawaddy Valley in N.W. Yunnan, China; ssp. *parcesquamata* in the Nujiang‐Salween River valley of N.W. Yunnan, China; ssp. *rubicundus* in the Lancang‐Mekong, Yuanjiang‐Red River valleys of C. Yunnan, China to N. Vietnam; ssp. *nigricauda* in the eastern margin of the upper Yangtze River watershed; ssp. *dealbatus* endemic to the Qinling Mountains; and ssp. *xamnuensis* endemic to N. Laos (Racheli and Bozano 2024). Several *Byasa* species can be found within the range of certain subspecies of *P. bootes*, which usually show limited phenotypes resembling one of a few ‘mainstream’ *Byasa* species. The mimicry between these two species has not been scientifically explored, apart from brief mentions in taxonomic literature (Nakae 2021).

To tackle this intriguing mimicry case in nature, the present study combined computer‐aided and human‐eyesight comparison to analyse the similarities between each subspecies of *P. bootes* and its sympatric *Byasa* species in China, where the diversity of both groups is the greatest (Racheli and Cotton 2010; Racheli and Bozano 2024). The purpose of such a design is to minimise the human‐mediated bias in traditional mimicry research (Bhuiyan et al. 2022), as well as to compensate for the missing part of animal‐visual recognition in research purely using computer models (Schnell et al. 2023). The findings of this study have the potential to facilitate our understanding of the mimicry between *P. bootes* and *Byasa* and provide new insights for future research on Batesian mimicry in butterflies.

## 2 Materials and Methods

### 2.1 Focal Taxon

This study focused on the taxon‐rich part of the distribution range of *Papilio bootes* and *Byasa* spp. in western China and analysed the five subspecies of *P. bootes* and 13 species of *Byasa*. The taxon names and their distribution ranges mainly followed Hu et al. (2023) and Racheli and Bozano (2024) (Table 1 and Figure 2).

**TABLE 1:** Distribution range of the five subspecies of *Papilio bootes* and the 13 species of *Byasa* in western China.

Taxon name	Distribution range
*Papilio bootes mindoni*	Dulongjiang‐Irrawaddy valley, west of Gaoligong Shan
*Papilio bootes parcesquamata*	Nujiang‐Salween valley, east of Gaoligong Shan
*Papilio bootes rubicundus*	Lancang‐Mekong and Yuanjiang‐Red River valleys
*Papilio bootes nigricauda*	Upper Yangtze watershed in Yunnan and Sichuan
*Papilio bootes dealbatus*	Qinling Mountains
*Byasa latreillei*	Confined to the west of Gaoligong Shan, including Dulongjiang valley of Yunnan and S.E. Tibet
*Byasa polla*	Confined to the west of Gaoligong Shan, and S.E. Tibet
*Byasa dasarada*	Mainly found in N.W. to *S. Yunnan*
*Byasa daemonius*	Upper Yangtze River valley in N.W. Yunnan
*Byasa plutonius*	Montane areas in of the Lancang‐Mekong, Nujiang‐Salween, and Dulong‐Irrawaddy valley of Yunnan; and subalpine montane areas in W. Sichuan to S. Shaanxi
*Byasa rhadinus*	A narrow‐ranged species endemic to the Cang Shan area in Dali, Yunnan
*Byasa crassipes*	Confined to the lowlands of W., S.W., S., and S.E. Yunnan
*Byasa genestieri*	Widely distributed in the montane areas east of the Gaoligong Shan to the S.E. part of Yunnan
*Byasa impediens*	Found in *S. Yunnan* to Guangxi, Sichuan, and Shaanxi
*Byasa nevilli*	Widely distributed throughout Yunnan, and part of Sichuan, Guizhou, and S.E. Tibet
*Byasa polyeuctes*	Widely distributed in S.W., S., C., and part of N. China south of Yanshan Mountains
*Byasa hedistus*	Widely distributed throughout Yunnan; also found in Sichuan
*Byasa confusus*	East margin of the Hengduan Mountains

### 2.2 Taxon Sampling

Specimens were collected from the distribution ranges of *P. bootes* and its sympatric *Byasa* species in China using insect nets. Captured individuals were killed by pinching the thorax and preserved in paper triangles, labelled with detailed collecting information, dried and stored at room temperature.

All specimens were spread in the laboratory and photographed using a Canon EOS 700D camera in a light box with a white background to standardise the exposure and white balance of each photograph, in order to minimise the differences induced by photographing in subsequent analyses. For each specimen, the dorsal and ventral sides were photographed using the same methods.

**FIGURE 1:**
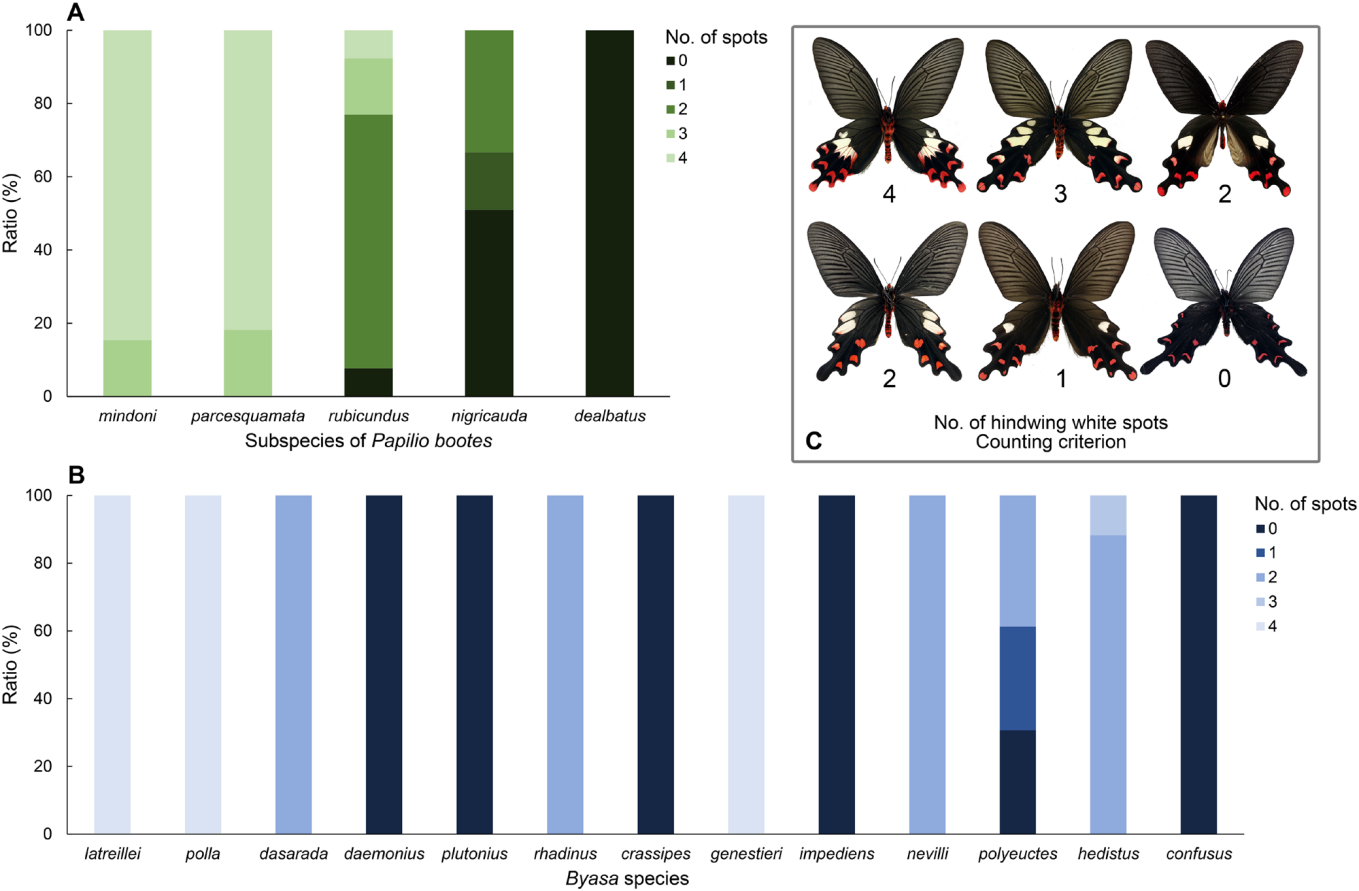
Percentages of the number of hindwing white spots in five subspecies of *Papilio bootes* (A) and 13 species of *Byasa* (B) in this study, with the upper right corner illustrating the counting criterion (C).

The hindwing white spots are highly variable across or even within taxa; therefore, the number of hindwing white spots was counted and plotted to show the percentage of each type and used in future analysis (Figure 1).

### 2.3 Computer‐Aided Morphological Comparison

This study employed the unsupervised deep learning algorithm SwAV (Caron et al. 2020) to extract morphological features from specimen photographs (Alam et al. 2024). Specifically, SwAV maps each butterfly image into a 2048‐dimension feature vector, capturing a range of features including wing shape, colour and hindwing markings (white discal and subapical spots, red submarginal spots and red tail spot), then analyses these feature vectors to enable a precise assessment of similarity between the focal taxa.

To obtain an efficient feature extractor, we first trained the SwAV model on a dataset comprising 19 butterfly species and a total of 251 images. The SwAV model was initialised with weights pretrained on ImageNet (Deng et al. 2009), a foundational visual database containing over 14 million hand‐annotated images across 20,000 everyday object categories. The pre‐training allows models to recognise basic visual patterns before specialising in domain‐specific tasks like butterfly morphology. The collected data set of 251 images was split into training, validation and test sets in an 8:1:1 ratio. After training, the feature extractor achieved a performance of 90.67% robustness on the test set.

Assume the data set *X* = {*X*_1_, *X*_2_, …, *X*_*n*_}, where *n* represented the number of samples. The feature vectors obtained after processing through the feature extractor can be denoted as *F* = *f*_1_, *f*_2_, …, *f*_*n*_. These extracted features were grouped according to their respective categories, and the average feature for each category is calculated. The mean feature vectors for different categories can be represented as $\bar{f}=\bar{f_{1}},\bar{f_{2}},\ldots ,\bar{f_{k}}$, where *k* denotes the number of butterfly species.

Cosine similarity was then employed to calculate the feature similarity between images of different butterfly species. *S*_*ij*_ represents the similarity between different species.

$$S_{\mathrm{ij}}=\frac{{\bar{f}}_{i}\cdot {\bar{f}}_{j}}{\left\|{\bar{f}}_{i}\right\|\left\|{\bar{f}}_{j}\right\|}$$

Where, $\bar{f}$ represents the average features of different butterfly species images from the sample *X* and denotes the feature similarity between species *i* and *j*. After calculating the similarity between different species, we created a heatmap to visualise the similarity among butterfly species. We mapped a set of Sankey diagrams based on the similarity values between *P. bootes* subspecies and *Byasa* species. The flows between taxa were represented by *Q*_*ij*_.

$$Q_{\mathrm{ij}}=-\log\left(1-S_{\mathrm{ij}}\right)$$

To visualise the distribution of the features, t‐SNE (Stochastic Neighbour Embedding) (Van der Maaten and Hinton 2008) is used for dimensional downscaling and mapped onto a two‐dimensional plane. The relationships between high‐dimensional sample points can be expressed as:

$$P_{j|i}=\frac{\exp\left(-∥f_{i}-f_{j}{∥}^{2}/2\delta_{i}^{2}\right)}{\sum_{k\neq i}\exp\left(-∥f_{i}-f_{k}{∥}^{2}/2\delta_{i}^{2}\right)}$$

and the position relationship of low‐dimensional sample points is expressed as:

$$q_{j|i}=\frac{\exp\left(-∥y_{i}-y_{j}{∥}^{2}\right)}{\sum_{k\neq i}\exp\left(-∥y_{i}-y_{k}{∥}^{2}\right)}$$

where, *y* represents the coordinates of different species in the low‐dimensional space.

Use Kullback–Leibler divergence (*KL*) (Kullback and Leibler 1951; Csiszar 1975) to measure the difference between the two probability distributions of *P*_*j*|*i*_ and *q*_*j*|*i*_:

$$C=\underset{i}{\sum \mathrm{KL}\left(P_{i}\|Q_{i}\right)}=\sum_{i}\sum_{j}p_{j|i}\log\frac{p_{j|i}}{q_{j|i}}$$

The gradient descent algorithm is used to optimise *y*, minimising *C* to achieve the goal of mapping high‐dimensional features to a low‐dimensional space. Finally, a multi‐dimension feature vector is downscaled to a three‐dimension vector and visualised on a 3D coordinate space. The data were computed using Python scripts and then visualised using Origin 2022b (OriginLab Corporation, Northampton, MA, USA) into a heatmap and 3D charts. Additional annotations to the Sankey diagram were added by Adobe Illustrator CS6 (Adobe Systems Inc., San Jose, CA, USA; licenced serial number 9229‐8586‐7036‐7176).

### 2.4 Human Eyesight Comparison

To complement the results of computer‐aided comparison, the present study implemented human eyesight comparison since human trichromatic vision is the closest among mammals to avian vision, although humans cannot perceive the ultraviolet portion of the spectrum like birds. An anonymous questionnaire survey with a Likert scale, a universal quantitative survey method to assess the feeling/attitude of participants, was utilised to collect the responses from all participants (Likert 1932). The basic information part of the survey questionnaire contains the participants' gender, age, education background and familiarity with butterfly taxonomy, in an attempt to detect bias in quality control. Control questions were designed to feature pairs of butterflies that are totally different from both *P. bootes* and *Byasa* spp. (e.g., *Graphium* and *Troides* species with extremely different wing shapes and colours), while the testing questions involved pairing different subspecies of *P. bootes* with different species of *Byasa* by rating the eyesight similarity using a 10‐point scale, with larger numbers indicating greater perceived similarity.

The survey questionnaires were distributed online via the platform Wenjuanxing (https://www.wjx.cn/), between March 20th and May 6th, 2024, using the Chinese social media WeChat to promote participation. After the survey, a total of 1006 questionnaires were collected. The results were exported in an Excel spreadsheet, and validation was performed to manually flag responses with inconsistencies in the control questions (i.e., different responses among three questions of the same designed answer), as well as the responses with contradictions in two or more sets of highly similar questions. After validation, 506 questionnaires were deemed valid and passed to subsequent analyses. Mean similarity scores of all testing questions were calculated and visualised into another set of Sankey diagrams.

Since historical nonsympatric mimicry is proved with evolutionary significance (Linares 1997; Pfennig and Mullen 2010), we generated two sets of Sankey diagrams from both the computer‐aided and human‐eyesight morphological comparisons. One set of the Sankey diagrams containing nonsympatric taxa was used to test possible historical mimicry, while the other set containing only sympatric taxa was used to show the current status.

## 3 Results

### 3.1 Computer‐Aided Morphological Comparison

**FIGURE 2:**
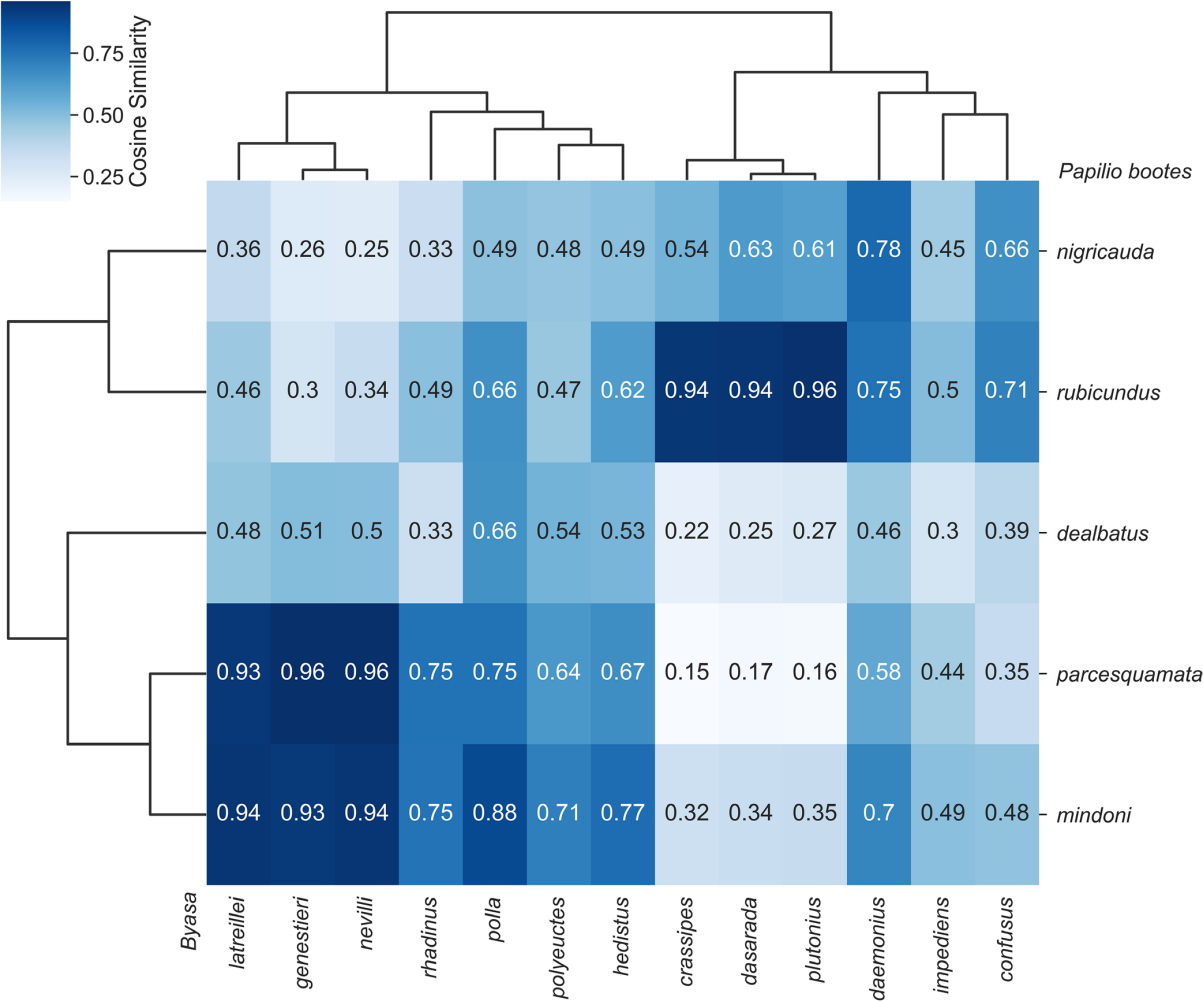
Heatmap of the similarity between *Papilio bootes* (*Y*‐axis) and 13 sympatric *Byasa* species (*X*‐axis) with hierarchical clustering.

This study analysed 260 specimens (Table 2), comprising five subspecies of *Papilio bootes* and thirteen *Byasa* species, revealing distinct geographical mimicry patterns (Figure 2). In the Dulongjiang‐Irrawaddy Valley in Northwest Yunnan, *P. bootes mindoni* exhibits strong morphological convergence with sympatric *B. latreillei* and *B. nevilli*, while showing minimal resemblance to all‐black species like *B. plutonius*. The Nujiang‐Salween Valley population (*P. bootes parcesquamata*) closely mirrors *B. nevilli* and *B. genestieri*, yet displays negligible similarity to local spotless species like *B. dasarada*.

**TABLE 2:** Collecting information and the number of samples of each taxon.

Taxon name	No. of specimens	Collection locality
*Papilio bootes mindoni*	13	Dehong, Yunnan
*Papilio bootes parcesquamata*	11	Fugong, Yunnan
*Papilio bootes rubicundus*	11	Kunming and Dali, Yunnan
*Papilio bootes nigricauda*	51	Ganzi and Ya'an, Sichuan
*Papilio bootes dealbatus*	11	Qinling Mountains, Shaanxi
*Byasa latreillei*	7	Dehong, Yunnan
*Byasa polla*	9	Dehong, Yunnan
*Byasa dasarada*	2	Dehong, Yunnan
*Byasa daemonius*	9	Diqing, Yunnan
*Byasa plutonius*	12	Dali, Yunnan
*Byasa rhadinus*	1	Dali, Yunnan
*Byasa crassipes*	4	Dehong, Yunnan
*Byasa genestieri*	18	Kunming, Yunnan
*Byasa impediens*	16	Ya'an, Sichuan
*Byasa nevilli*	6	Kunming, Yunnan
*Byasa polyeuctes*	69	Dehong, Dali, and Kunming, Yunnan; Ya'an, Sichuan
*Byasa hedistus*	7	Dali and Kunming, Yunnan
*Byasa confusus*	6	Ya'an, Sichuan

Moving southward through the Lancang‐Mekong and Yuanjiang‐Red River Valleys, *P. bootes rubicundus* demonstrates pronounced alignment with *B. plutonius* and *B. crassipes* , contrasting sharply with its dissimilarity to white‐spotted *Byasa* species. Along the eastern Yangtze fringe, *P. bootes nigricauda* shows greater morphological diversity than other subspecies, with strongest affinities for spotless models like *B. daemonius* and *B. confusus* . The Qinling Mountains endemic (*P. bootes dealbatus*) presents relatively low mimicry specialisation, though maintains measurable connections to *B. polyeuctes*.

**FIGURE 3:**
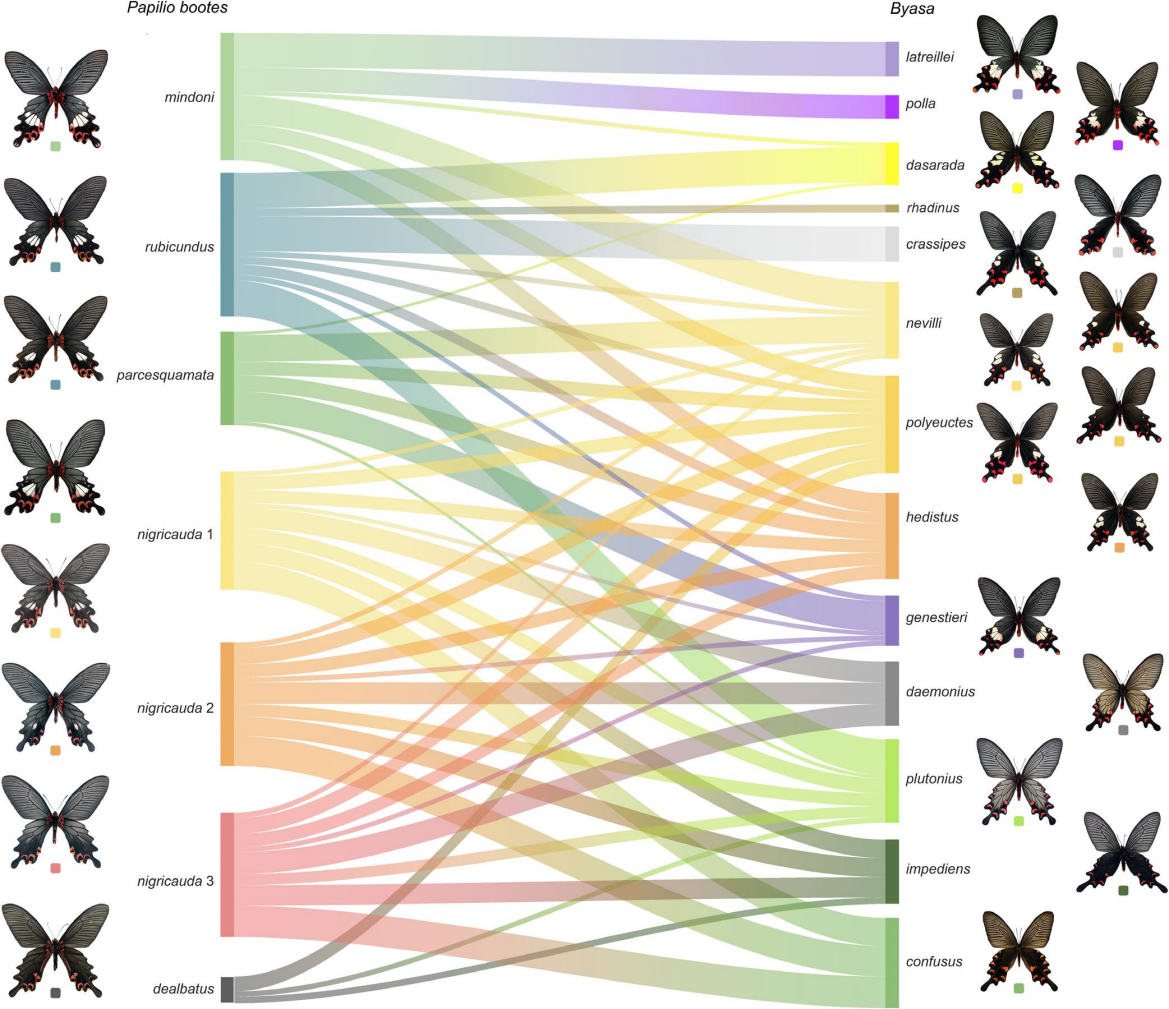
Sankey diagram of the computer‐aided similarity between five subspecies of *Papilio bootes* and 13 *Byasa* species (currently sympatric only). The width of connecting bands represents the degree of similarities, with broader bands indicating higher similarity. Butterflies are colour‐coded, with each colour corresponding to a specific taxon group.

These regional partnerships are visually synthesised in the Sankey diagram (Figure 3), which maps morphological affinity flows between sympatric taxa. *P. bootes mindoni* channels robust similarity towards *B. latreillei* and *B. polla*, while *P. bootes parcesquamata* shows dominant linkages to *B. nevilli* and *B. genestieri* in its restricted range. For *P. bootes rubicundus*, substantive flows connect to multiple *Byasa* species including *B. polyeuctes* and *B. hedistus*, whereas *P. bootes nigricauda* exhibits focused convergence with spotless *B. daemonius* and *B. plutonius*. Notably, *P. bootes dealbatus* displays selective affinity for *B. polyeuctes* despite overall weak mimicry associations. When taking nonsympatric taxa into the analysis, the Sankey diagram shows a more evenly distributed channel connections between the darker or all‐black subspecies of *P. bootes* (*rubicundus*, two types of *nigricauda* and *dealbatus*) and *Byasa* species, while the *P. bootes* with large hindwing white spots are more connected to the most similar *Byasa* species mentioned above (Figure S1).

**FIGURE 4:**
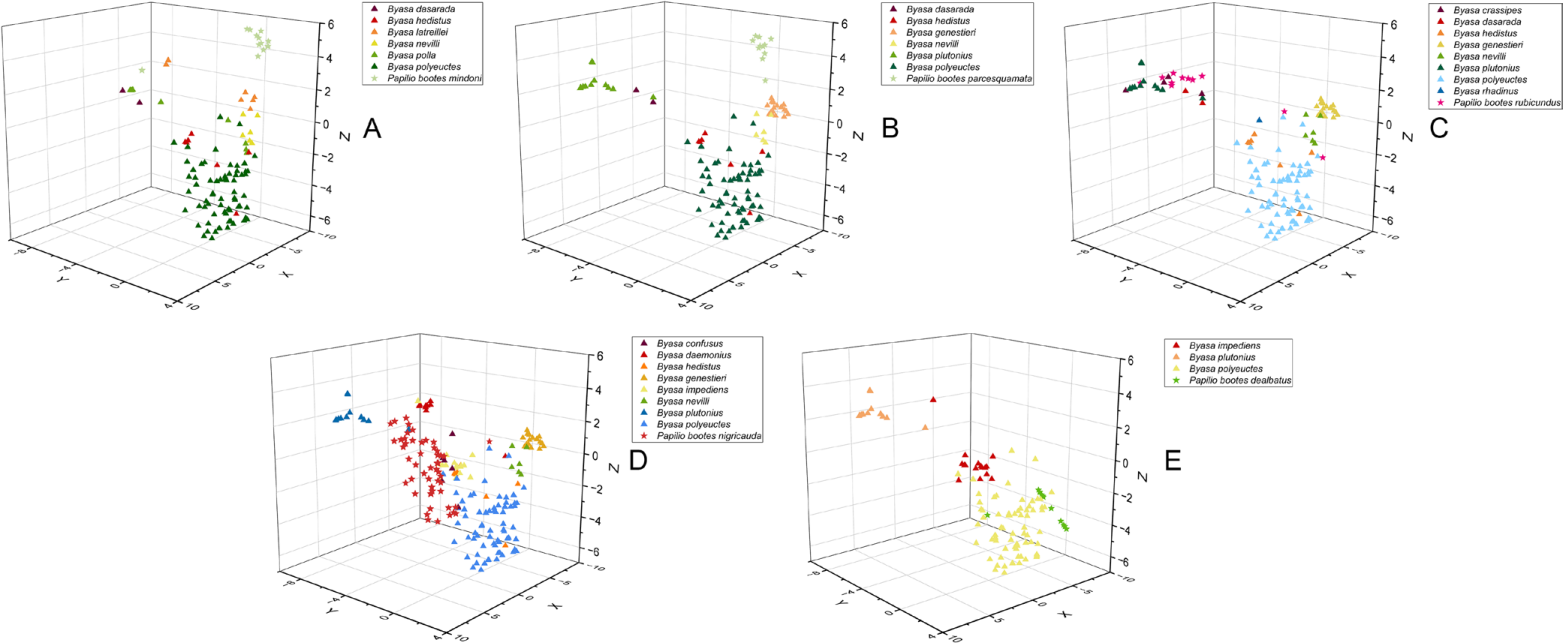
3‐D t‐SNE scatter plots showing the similarity between five *P. bootes* subspecies and their sympatric *Byasa* species. Pentagrams denote *Papilio bootes* and triangles represent *Byasa* in each plot.

Cross‐regionally, 3D scatter plots (Figure 4) confirm tighter morphological clustering in four subspecies compared with the variable *P. bootes nigricauda*. *Byasa polyeuctes* emerges as a generalist mimic, maintaining consistent intermediate similarity with all *P. bootes* subspecies across their ranges—unlike specialists such as *B. crassipes* which show strong geographical limitation. Phylogenetic analysis further contextualises these relationships, dividing *Byasa* into two primary clades: white‐spotted species (e.g., *B. nevilli*, *B. polyeuctes*) and predominantly spotless species (e.g., *B. dasarada*, *B. plutonius*), with mimicry partnerships consistently aligning with these evolutionary groupings within each geographical region.

### 3.2 Human Eyesight Comparison

**FIGURE 5:**
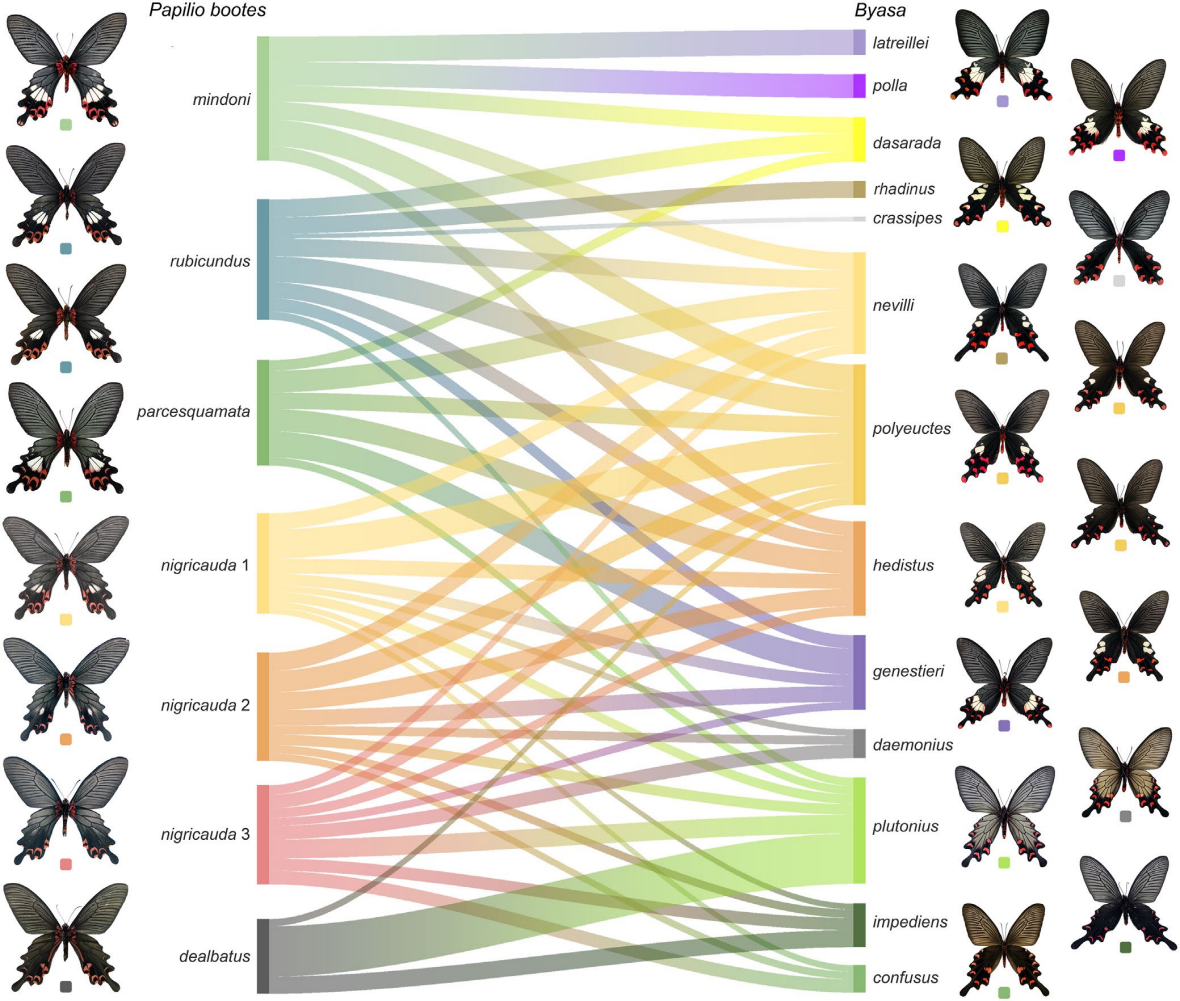
Sankey diagram based on the survey questionnaires on the similarity between five subspecies of *Papilio bootes* and 13 *Byasa* species (currently sympatric only); the band widths indicate morphological similarities. Butterfly taxa are colour‐coded accordingly.

Questionnaire responses from 506 participants possessing limited taxonomic familiarity, yielded reliable perceptual data on mimicry similarity. The Sankey diagram (Figure 5) analysis revealed distinct geographical patterns in human perception. In the Dulongjiang‐Irrawaddy Valley in Northwest Yunnan, *P. bootes mindoni* was perceived as most similar to *B. latreillei* , *B. polla* and *B. polyeuctes*, with secondary resemblance to *B. dasarada* and *B. nevilli*, while showing minimal perceptual alignment with *B. hedistus*.

Within the Nujiang‐Salween Valley of Northwest Yunnan, *P. bootes parcesquamata* exhibited strongest visual affinity to *B. genestieri*, followed by *B. nevilli*, *B. polyeuctes* and *B. hedistus*, contrasting with its weakest perceived similarities to *B. dasarada* and *B. plutonius*. Further south in the Lancang‐Mekong and Red River Valleys in Central Yunnan, *P. bootes rubicundus* demonstrated highest perceptual correspondence with *B. polyeuctes*, followed by *B. dasarada*, *B. rhadinus*, *B. nevilli*, *B. hedistus* and *B. genestieri*, though it showed minimal visual alignment with *B. crassipes* and *B. plutonius*.

The Qinling Mountains endemic *P. bootes dealbatus* displayed strongest perceived resemblance to *B. plutonius* with secondary affinity to *B. impediens*, while showing negligible visual similarity to *B. polyeuctes*. Notably within the morphologically variable Upper Yangtze population (*P. bootes nigricauda*), human perception varied considerably by colour morph: white‐spotted individuals showed strong alignment with *B. polyeuctes* (or with *B. nevilli* and *B. hedistus* in one specimen) but minimal resemblance to spotless species like *B. daemonius*, *B. impediens* and *B. confusus* ; in contrast, completely dark nonspotted forms demonstrated highest similarity to *B. plutonius* with weakest correspondence to white‐spotted *B. nevilli* and *B. genestieri*.

When taking nonsympatric taxa into the analysis, the Sankey diagram also shows a more evenly distributed channel connections between the darker or all‐black subspecies of *P. bootes* (*rubicundus* and two types of *nigricauda*) and *Byasa* species, while the *P. bootes* with large hindwing white spots and the Qinling Mountain endemic *dealbatus* are more connected to the most similar *Byasa* species mentioned above (Figure S2).

## 4 Discussion

The present study quantitatively demonstrated that *Papilio bootes* has a mimetic strategy of ‘targeting’ its sympatric *Byasa* species, especially in terms of hindwing white spots. It became clear that in West Yunnan, both subspecies of *P. bootes*, namely *mindoni* and *parcesquamata*, primarily mimic *B. latreillei* , *B. polla* and *B. genestieri*, the only three species with up to four hindwing white spots. Similarly, in the Qinling Mountains, *P. bootes dealbatus* specifically mimics *Byasa* without any hindwing white spots, *B. impediens*, *B. plutonius* and black *B. polyeuctes* (Figures 3, 5, S1 and S2). Between the two extremes, the other two subspecies of *P. bootes*, *rubicundus* and *nigricauda*, showed a more ‘flexible’ way of mimicking, which ranged from the four‐spotted *B. genestieri* in the western part to the spotless *Byasa* species in the northeastern part (Figures 3, 5, S1 and S2).

*B. polyeuctes* is interesting among several mimicry models of ssp. *rubicundus* and ssp. *nigricauda*. Primarily, *B. polyeuctes* has two white spots on the hindwing, but the number and size of these spots are highly variable. Wu and Hsu (2007) and Racheli and Cotton (2010) showed that in South and West Yunnan, the hindwing white spots of *B. polyeuctes* are larger than those in other parts of the Chinese mainland, while in the northern part of Yunnan, as well as the entire Sichuan and Shaanxi, the hindwing white spots of *B. polyeuctes* are reduced in both numbers and size to a complete black hindwing type. The variability in the number of hindwing discal white spots (0–2) in *B. polyeuctes* is evidently reflected by the morphological diversity found in ssp. *rubicundus* and ssp. *nigricauda* from the south to the north in their distribution range (Figures 3, 5, S1 and S2), forming a morphological gradient. It is worth mentioning that there are certain similarities between ssp. *mindoni* and ssp. *parcesquamata* and *B. polyeuctes* recognised by both computer‐aided and human eyesight comparisons, which can be explained by the larger hindwing white spots of sympatric *B. polyeuctes*.

Phylogenetic research of Papilionidae showed that *P. bootes* diverged quite recently in time (1.09–3.61 Ma, 95% HPD) (Condamine et al. 2023) compared to that of genus *Byasa* (12.26–23.21 Ma, 95% HPD), despite a few taxa diverging very recently (e.g., *B. dasarada*, *B. hedistus*, and *B. confusus* ) (S.‐J. Hu. B. J. Lafon, Z.‐B. Xu, M. Tang, A. M. Cotton, Y.‐Q. Jia, X. Zhang, S.‐X. Ge, K. Duan, F. L. Condamine unpublished data; Figure S3). Hence, it is logical to assume that the mimetic traits of *P. bootes* were accumulated through natural selection, that the morphological traits representing unpalatable images were gained over time to enhance the fitness of each population (or subspecies) (Mallet and Joron 1999; Mauro 2002; Caro et al. 2016; Ruxton et al. 2019). The variable *B. polyeuctes* diverged 11.49 Ma (95% HPD: 7.57–15.59 Ma) in the Hengduan Mountains of southwestern China, where two spotless species (*B. daemonius* and *B. crassipes* ) and three four‐spotted species ( *B. latreillei* , *B. polla* and *B. genestieri*) originated before it since 14.19 Ma (95% HPD: 9.47–19.16 Ma) (S.‐J. Hu. B. J. Lafon, Z.‐B. Xu, M. Tang, A. M. Cotton, Y.‐Q. Jia, X. Zhang, S.‐X. Ge, K. Duan, F. L. Condamine unpublished data). Therefore, it is highly likely that the larger hindwing white spots of *B. polyeuctes* are an ancestral character to increase its fitness among those four‐spotted species, and the spot‐reducing character is a subsequent adaptive trait after expanding its range into the areas dominated by other spotless species like *B. impediens* and *B. plutonius*. In this case, the possible Müllerian mimicry within *Byasa* might have shaped the spot variability of *B. polyeuctes* (Figures 2, 4), similar to the mimicry among different *Heliconius* species (Pérochon et al. 2025). In the transition area between the four‐spotted and the spotless *Byasa* species, the variability of *B. polyeuctes* plays a bridge to mingle the *P. bootes* and the various sympatric *Byasa* species with different morphological characters.

Although both computer‐aided and human eyesight comparison can effectively assist theoretical research on butterfly mimicry, the readers must bear in mind that predators' visions in the real world are different. For instance, birds with UV vision are better capable of distinguishing yellow and white areas on the wings of *Heliconius* species compared to those without it. This perceptual advantage helps maintain effective mimicry signals, enhancing their mimicry effectiveness (Dell'Aglio et al. 2018). Future studies could incorporate UV spectrum to obtain a more comprehensive understanding of butterfly mimicry. Moreover, field simulations using dummy insects with novo material could also benefit this research field by obtaining first‐hand predating/attacking data (Roslin et al. 2017; Pan et al. 2021; Nimalrathna et al. 2023).

Overall, this study corroborates the mimicry of *P. bootes* using two complementary analytical approaches: image similarity analysis and questionnaire‐based perception surveys. The image similarity analysis quantitatively assessed the degree of resemblance between *P. bootes* and *Byasa* species, while the survey evaluated the ability of human observers to distinguish between them. These results suggest that mimicry in *P. bootes* operates not only at the morphological level but also influences recognition at the level of visual perception (Mallet and Joron 1999; Kunte 2009). The evolution of mimetic traits in *P. bootes* is shaped not only by morphological convergence but also by complex ecological factors. Its similarity to *Byasa* species likely results from long‐term selective pressures rather than close phylogenetic relatedness (Gilbert 2005; Wu and Hsu 2007). This finding aligns with classical Batesian mimicry theory and further emphasises the critical role of environmental factors in driving mimicry evolution (Seymoure 2016).

## Author Contributions

**Yuan‐Rui‐Xue Xie:** formal analysis (lead), investigation (lead), methodology (equal), visualization (equal), writing – original draft (lead), writing – review and editing (equal). **Zhi‐Xing Ding:** formal analysis (equal), methodology (equal). **Adam M. Cotton:** resources (equal), writing – review and editing (equal). **Zhen‐Bang Xu:** investigation (equal), methodology (equal). **Yue Pan:** investigation (equal), methodology (equal). **Yi‐Ting Lin:** investigation (equal), methodology (equal). **Shao‐Ji Hu:** conceptualization (lead), funding acquisition (lead), methodology (equal), resources (equal), supervision (equal), writing – review and editing (lead).

## Conflicts of Interest

The authors declare no conflicts of interest.

## Supporting information


**Figure S1:** Sankey diagram of the computer‐aided similarity between five subspecies of *Papilio bootes* and 13 *Byasa* species (including non‐sympatric). The width of connecting bands represent the degree of similarities, with broader bands indicating higher similarity. Butterflies are colour‐coded, with each colour corresponding to a specific taxon group.

**Figure S2:** Sankey diagram based on the survey questionnaires on the similarity between five subspecies of *Papilio bootes* and 13 *Byasa* species (including nonsympatric), the band widths indicate morphological similarities. Butterfly taxa are colour‐coded accordingly.

**Figure S3:** Unpublished Bayesian dated tree and biogeographic history of *Byasa*. Node values represent median divergence times with coloured bars representing the 95% HPD. Squares on each node represent the estimated ancestral areas, while circles at the end of each branch represent current species distribution areas. Along branches, triangles indicate dispersal from ancestral areas to descendant distribution areas, daggers indicate extirpation from ancestral areas, and the colours of all these symbols mirror those on the biogeographic map in the top‐left corner. Explosion symbols represent vicariance events.

**Data S1:** ece372369‐sup‐0004‐SupplementaryTables.zip.

## Data Availability

The data supporting this study are openly available in Supporting Information.

## References

[ece372369-bib-0001] Alam, N. U. , E. H. Bahadur , A. K. M. Masum , F. M. Noori , and Z. Uddin . 2024. “SwAV‐Driven Diagnostics: New Perspectives on Grading Diabetic Retinopathy From Retinal Photography.” Frontiers in Robotics and AI 11: 1445565. 10.3389/frobt.2024.1445565. 39346742 PMC11427755

[ece372369-bib-0002] Anderson, B. , and M. L. de Jager . 2020. “Natural Selection in Mimicry.” Biological Reviews 95, no. 2: 291–304. 10.1111/brv.12564. 31663254

[ece372369-bib-0003] Basu, D. N. , V. Bhaumik , and K. Kunte . 2023. “The Tempo and Mode of Character Evolution in the Assembly of Mimetic Communities.” PNAS 120: e2203724120. 10.1073/pnas.2203724120. 36577073 PMC9910590

[ece372369-bib-0004] Bhuiyan, T. , R. M. Carney , and S. Chellappan . 2022. “Artificial Intelligence Versus Natural Selection: Using Computer Vision Techniques to Classify Bees and Bee Mimics.” iScience 25, no. 9: 104924. 10.1016/j.isci.2022.104924. 36060073 PMC9437854

[ece372369-bib-0005] Bonner, J. T. 1988. The Evolution of Complexity: By Means of Natural Selection, 272. Princeton University Press.

[ece372369-bib-0006] Brower, J. V. Z. 1958. “Experimental Studies of Mimicry in Some North American Butterflies: Part II. *Battus philenor* and *Papilio troilus*, *P. polyxenes* and *P. glaucus* .” Evolution 12, no. 2: 123–136. 10.1111/j.1558-5646.1958.tb02939.x.

[ece372369-bib-0007] Caro, T. , T. N. Sherratt , and M. Stevens . 2016. “The Ecology of Multiple Colour Defences.” Evolutionary Ecology 30: 797–809. 10.1007/s10682-016-9854-3.

[ece372369-bib-0008] Caron, M. , I. Misra , J. Mairal , P. Goyal , P. Bojanowski , and A. Joulin . 2020. “Unsupervised Learning of Visual Features by Contrasting Cluster Assignments.” Paper presented at the 34th International Conference on Neural Information Processing System.Vancouver, BC, Canada. 9912–9924.

[ece372369-bib-0009] Chai, P. 1996. “Butterfly Visual Characteristics and Ontogeny of Responses to Butterflies by a Specialized Tropical Bird.” Biological Journal of the Linnean Society 59, no. 1: 37–67. 10.1006/bijl.1996.0053.

[ece372369-bib-0010] Clarke, C. , and P. M. Sheppard . 1972. “The Genetics of the Mimetic Butterfly *Papilio polytes* L.” Philosophical Transactions of the Royal Society of London B 263, no. 855: 431–458. 10.1098/rstb.1972.0006. 4402450

[ece372369-bib-0011] Condamine, F. L. , R. Allio , E. L. Reboud , et al. 2023. “A Comprehensive Phylogeny and Revised Taxonomy Illuminate the Origin and Diversification of the Global Radiation of *Papilio* (Lepidoptera: Papilionidae).” Molecular Phylogenetics and Evolution 183: 107758. 10.1016/j.ympev.2023.107758. 36907224

[ece372369-bib-0012] Csiszar, I. 1975. “I‐Divergence Geometry of Probability Distributions and Minimization Problems.” Annals of Probability 3, no. 1: 146–158. 10.1214/aop/1176996454.

[ece372369-bib-0013] Dell'Aglio, D. D. , J. Troscianko , W. O. McMillan , M. Stevens , and C. D. Jiggins . 2018. “The Appearance of Mimetic *Heliconius* Butterflies to Predators and Conspecifics.” Evolution 72, no. 10: 2156–2166. 10.1111/evo.13583. 30129174 PMC6221148

[ece372369-bib-0014] Deng, J. , W. Dong , R. Socher , K. Li , and F. F. Li . 2009. “ImageNet: A Large‐Scale Hierarchical Image Database.” Paper presented at the 2009 IEEE Conference on Computer Vision and Pattern Recognition. Miami, FL, USA. 248–255.

[ece372369-bib-0015] Futahashi, R. , and H. Fujiwara . 2008. “Juvenile Hormone Regulates Butterfly Larval Pattern Switches.” Science 319, no. 5866: 1061. 18292334 10.1126/science.1149786

[ece372369-bib-0016] Gilbert, F. 2005. “The Evolution of Imperfect Mimicry.” Paper presented at the Proceedings of the Royal Entomological Society's 22nd Symposium. UK: CABI Publishing.

[ece372369-bib-0017] Hu, S. J. , A. M. Cotton , G. Lamas , K. Duan , and X. Zhang . 2023. “Checklist of Yunnan Papilionidae (Lepidoptera: Papilionoidea) With Nomenclatural Notes and Descriptions of New Subspecies.” Zootaxa 5362, no. 1: 1–69. 10.11646/zootaxa.5362.1.1. 38220735

[ece372369-bib-0018] Kristensen, N. P. , M. J. Scoble , and O. L. E. Karsholt . 2007. “Lepidoptera Phylogeny and Systematics: The State of Inventorying Moth and Butterfly Diversity.” Zootaxa 1668, no. 1: 699–747. 10.11646/Zootaxa.1668.1.30.

[ece372369-bib-0019] Kronforst, M. R. , and R. Papa . 2015. “The Functional Basis of Wing Patterning in *Heliconius* Butterflies: The Molecules Behind Mimicry.” Genetics 200, no. 1: 1–19. 10.1534/genetics.114.172387. 25953905 PMC4423356

[ece372369-bib-0020] Kullback, S. , and R. A. Leibler . 1951. “On Information and Sufficiency.” Annals of Mathematical Statistics 22, no. 1: 79–86. 10.1214/aoms/1177729694.

[ece372369-bib-0021] Kunte, K. 2009. “The Diversity and Evolution of Batesian Mimicry in *Papilio* Swallowtail Butterflies.” Evolution 63, no. 10: 2707–2716. 10.1111/j.1558-5646.2009.00752.x. 19552738

[ece372369-bib-0022] Likert, R. 1932. “A Technique for the Measurement of Attitudes.” Archives of Psychology 22, no. 140: 5–55.

[ece372369-bib-0023] Linares, M. 1997. “The Ghost of Mimicry Past: Laboratory Reconstitution of an Extinct Butterfly ‘Race’.” Heredity 78: 628–635. 10.1038/hdy.1997.102.

[ece372369-bib-0024] Mallet, J. 2008. “Wallace and the Species Concept of the Early Darwinians.” In Natural Selection and Beyond: The Intellectual Legacy of Alfred Russell Wallace, edited by C. R. Smith and G. W. Beccaloni , 1–9. Oxford University Press.

[ece372369-bib-0025] Mallet, J. , and L. E. Gilbert . 1995. “Why Are There So Many Mimicry Rings? Correlations Between Habitat, Behaviour and Mimicry in *Heliconius* Butterflies.” Biological Journal of the Linnean Society 55, no. 2: 159–180. 10.1006/bijl.1995.0034.

[ece372369-bib-0026] Mallet, J. , and M. Joron . 1999. “Evolution of Diversity in Warning Color and Mimicry: Polymorphisms, Shifting Balance, and Speciation.” Annual Review of Ecology, Evolution, and Systematics 30: 201–233. 10.1146/annurev.ecolsys.30.1.201.

[ece372369-bib-0027] Mauro, G. 2002. Palatability and Anti‐Predation Strategies in Costa Rican Butterflies, September 2002, 487. Tropical Ecology and Conservation [Monteverde Institute]. https://digitalcommons.usf.edu/tropical_ecology/487.

[ece372369-bib-0028] Nakae, M. 2021. Papilionidae of the World, 335. Roppon‐Ashi.

[ece372369-bib-0029] Nijhout, H. F. 1991. The Development and Evolution of Butterfly Wing Patterns, 322. Smithsonian Institution.

[ece372369-bib-0030] Nijhout, H. F. 2001. “Elements of Butterfly Wing Patterns.” Journal of Experimental Zoology 291, no. 3: 213–225. 10.1002/jez.1099. 11598911

[ece372369-bib-0031] Nimalrathna, T. S. , I. D. Solina , A. M. Mon , et al. 2023. “Estimating Predation Pressure in Ecological Studies: Controlling Bias Imposed by Using Sentinel Plasticine Prey.” Entomologia Experimentalis et Applicata 171, no. 1: 56–67. 10.1111/eea.13249.

[ece372369-bib-0032] Nishida, R. , and H. Fukami . 1989. “Ecological Adaptation of an Aristolochiaceae‐Feeding Swallowtail Butterfly, *Atrophaneura alcinous*, to Aristolochic Acids.” Journal of Chemical Ecology 15: 2549–2563. 10.1007/BF01014731. 24271597

[ece372369-bib-0033] Nishikawa, H. , M. Iga , J. Yamaguchi , et al. 2013. “Molecular Basis of Wing Coloration in a Batesian Mimic Butterfly, Papilio Polytes.” Scientific Reports 3, no. 1: 3184. 10.1038/srep03184. 24212474 PMC3822385

[ece372369-bib-0034] Outomuro, D. , P. Ángel‐Giraldo , A. Corral‐Lopez , and E. Realpe . 2016. “Multitrait Aposematic Signal in Batesian Mimicry.” Evolution 70, no. 7: 1596–1608. 10.1111/evo.12963. 27241010

[ece372369-bib-0035] Pan, X. , T. Mizuno , K. Ito , et al. 2021. “Assessing Temporal Dynamics of Predation and Effectiveness of Caterpillar Visual Defense Using Sawfly Larval Color and Resting Posture as a Model.” Insect Science 28, no. 6: 1800–1815. 10.1111/1744-7917.12884. 33205542

[ece372369-bib-0036] Pérochon, E. , N. Rosser , K. Kozak , et al. 2025. “Müllerian Mimicry in Neotropical Butterflies: One Mimicry Ring to Bring Them All, and in the Jungle Bind Them.” *bioRxiv*: 1–26. 10.1101/2025.01.30.635679.

[ece372369-bib-0037] Pfennig, D. W. , and S. P. Mullen . 2010. “Mimics Without Models: Causes and Consequences of Allopatry in Batesian Mimicry Complexes.” Proceedings of the Royal Society B: Biological Sciences 1694: 2577–2585. 10.1098/rspb.2010.0586 PMC2982051 20484238

[ece372369-bib-0038] Prudic, K. L. , J. C. Oliver , and F. A. H. Sperling . 2007. “The Signal Environment Is More Important Than Diet or Chemical Specialization in the Evolution of Warning Coloration.” PNAS 104, no. 49: 19381–19386. 10.1073/pnas.0705478104. 18029450 PMC2148298

[ece372369-bib-0039] Racheli, T. , and G. C. Bozano . 2024. “Guide to the Butterflies of the Palearctic Region.” In Papilionidae Part V. Subfamily Papilionidae, Tribe Papilionini, Genus Papilio, 109. Omnes Artes.

[ece372369-bib-0040] Racheli, T. , and A. M. Cotton . 2010. “Guide to the Butterflies of the Palearctic Region.” In Papilionidae Part II. Subfamily Papilionidae, Tribe Troidini, 86. Omnes Artes.

[ece372369-bib-0041] Roslin, T. , B. Hardwick , V. Novotny , et al. 2017. “Higher Predation Risk for Insect Prey at Low Latitudes and Elevations.” Science 356, no. 6339: 742–744. 10.1126/science.aaj1631. 28522532

[ece372369-bib-0042] Ruxton, G. D. , W. L. Allen , T. N. Sherratt , and M. P. Speed . 2019. Avoiding Attack: The Evolutionary Ecology of Crypsis, Aposematism, and Mimicry. 2nd ed, xi+278. Oxford University Press.

[ece372369-bib-0043] Schnell, A. E. , M. Leemans , K. Vinken , and H. O. de Beeck . 2023. “A Computationally Informed Comparison Between the Strategies of Rodents and Humans in Visual Object Recognition.” eLife 12: RP87719. 10.7554/eLife.87719.3. 38079481 PMC10712954

[ece372369-bib-0044] Seymoure, B. M. 2016. “Heliconius in a New Light: The Effects of Light Environments on Mimetic Coloration, Behavior, and Visual Systems.” Arizona State University.

[ece372369-bib-0045] Song, N. , and A. P. Liang . 2009. “Complete Mitochondrial Genome of the Small Brown Planthopper, *Laodelphax striatellus* (Delphacidae: Hemiptera), With a Novel Gene Order.” Zoological Science 26, no. 12: 851–860. 10.2108/zsj.26.851. 19968473

[ece372369-bib-0046] Turner, J. R. G. 1984. “Mimicry: The Palatability Spectrum and Its Consequences.” In The Biology of Butterflies (Symposia of the Royal Entomological Society of London, 11), edited by R. I. Vane‐Wright and P. R. Ackery , 141–161. Academic Press.

[ece372369-bib-0047] Van der Maaten, L. , and G. Hinton . 2008. “Visualizing Data Using t‐SNE.” Journal of Machine Learning Research 9, no. 11: 2579–2605.

[ece372369-bib-0048] Wu, C. S. , and Y. F. Hsu . 2007. Butterflies of China, 2036. Strait Publishing House.

