# Supplementary figures and images for: Seeing Through the Mimicry of Papilio bootes by Combining Computer‐Aided and Human Eyesight Morphological Comparisons
Source: Ecol Evol. 2025 Oct 30;15(11):e72369. doi: 10.1002/ece3.72369 (PMC12572630; doi:10.1002/ece3.72369)

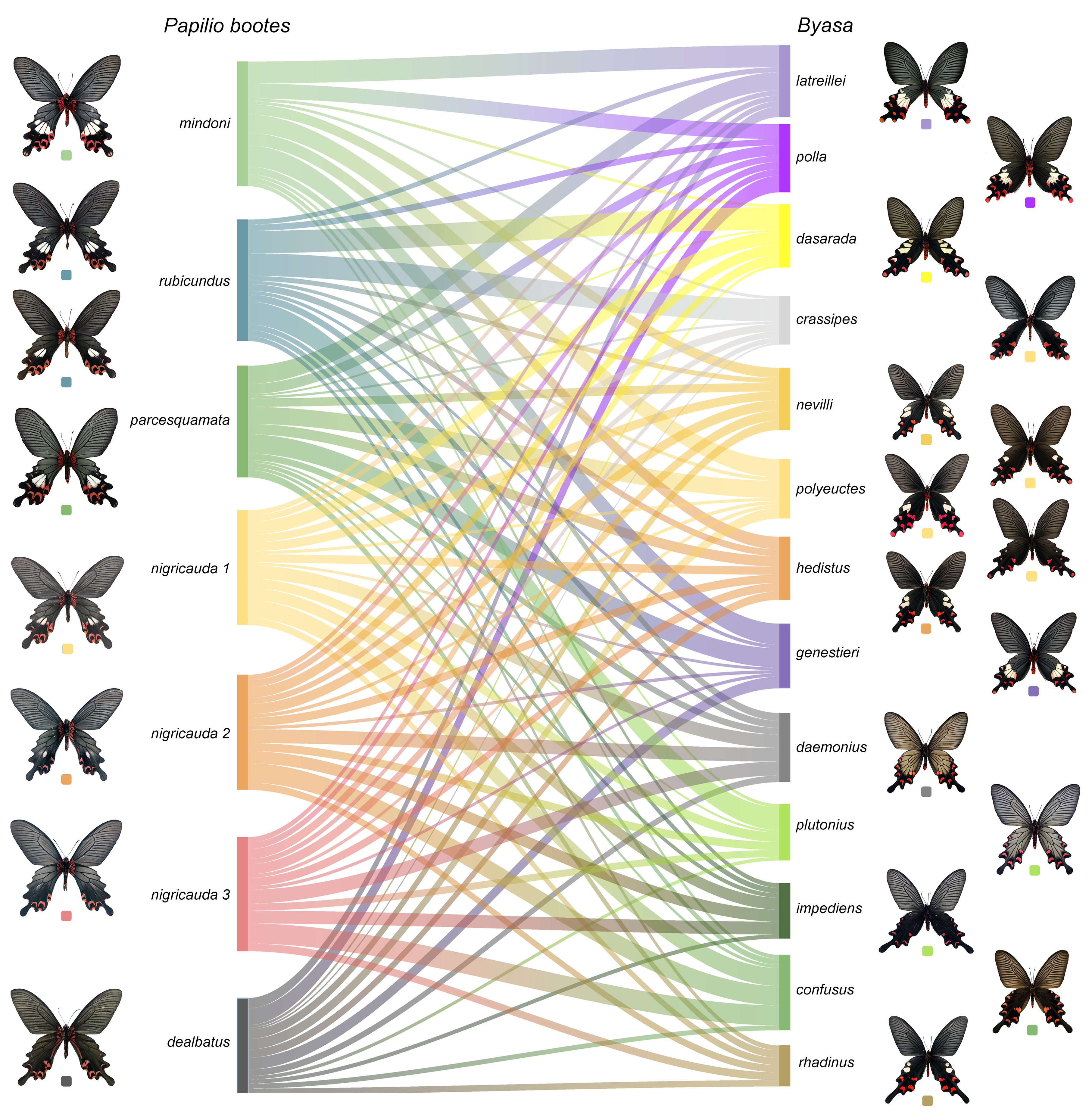

Supplement: Supplementary file 1 — Figure S1: Sankey diagram of the computer‐aided similarity between five subspecies of Papilio bootes and 13 Byasa species (including non‐sympatric). The width of connecting bands represent the degree of similarities, with broader bands indicating higher similarity. Butterflies are colour‐coded, with each colour corresponding to a specific taxon group. [file ECE3-15-e72369-s003.tif]

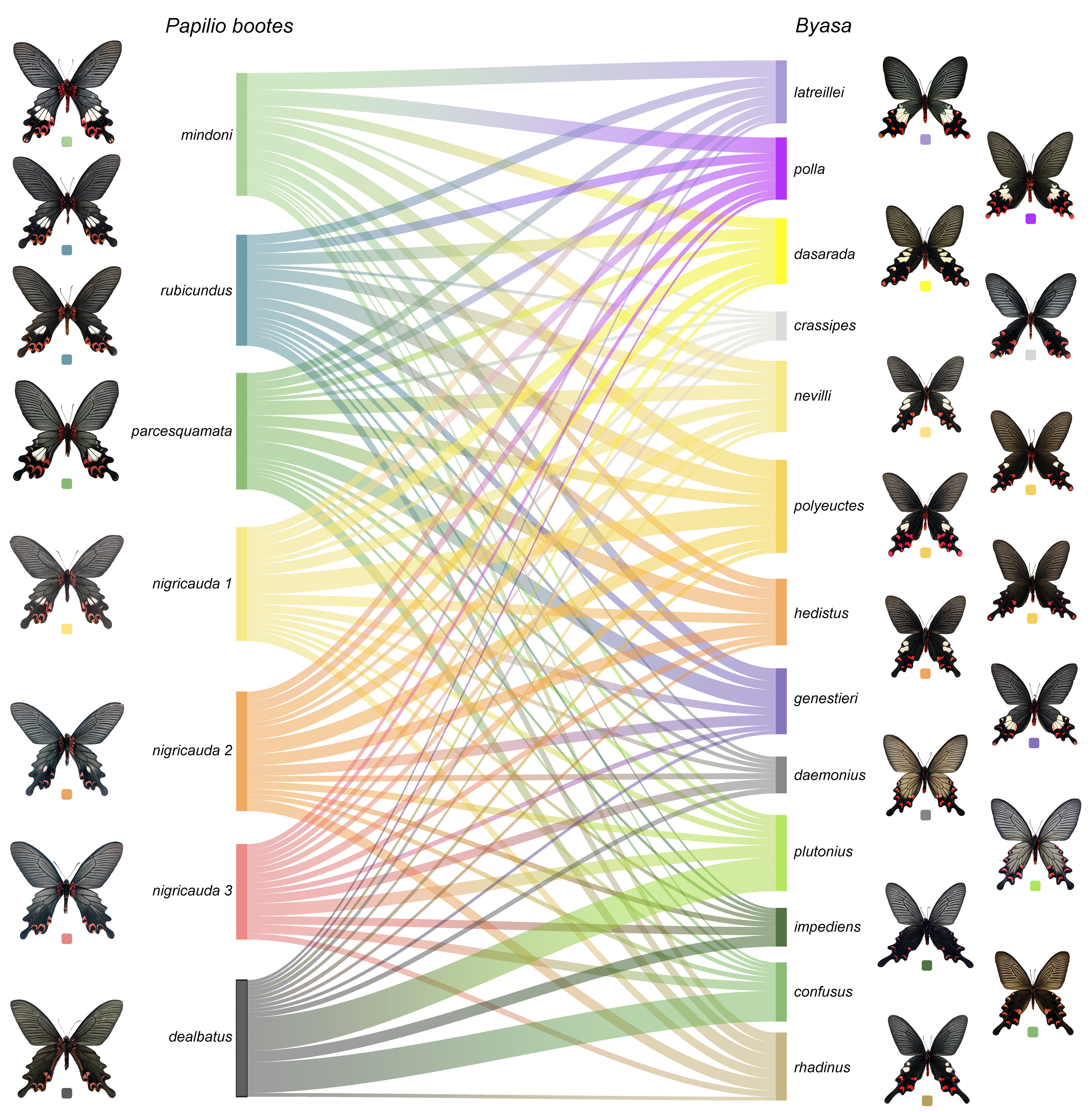

Supplement: Supplementary file 2 — Figure S2: Sankey diagram based on the survey questionnaires on the similarity between five subspecies of Papilio bootes and 13 Byasa species (including nonsympatric), the band widths indicate morphological similarities. Butterfly taxa are colour‐coded accordingly. [file ECE3-15-e72369-s001.tif]

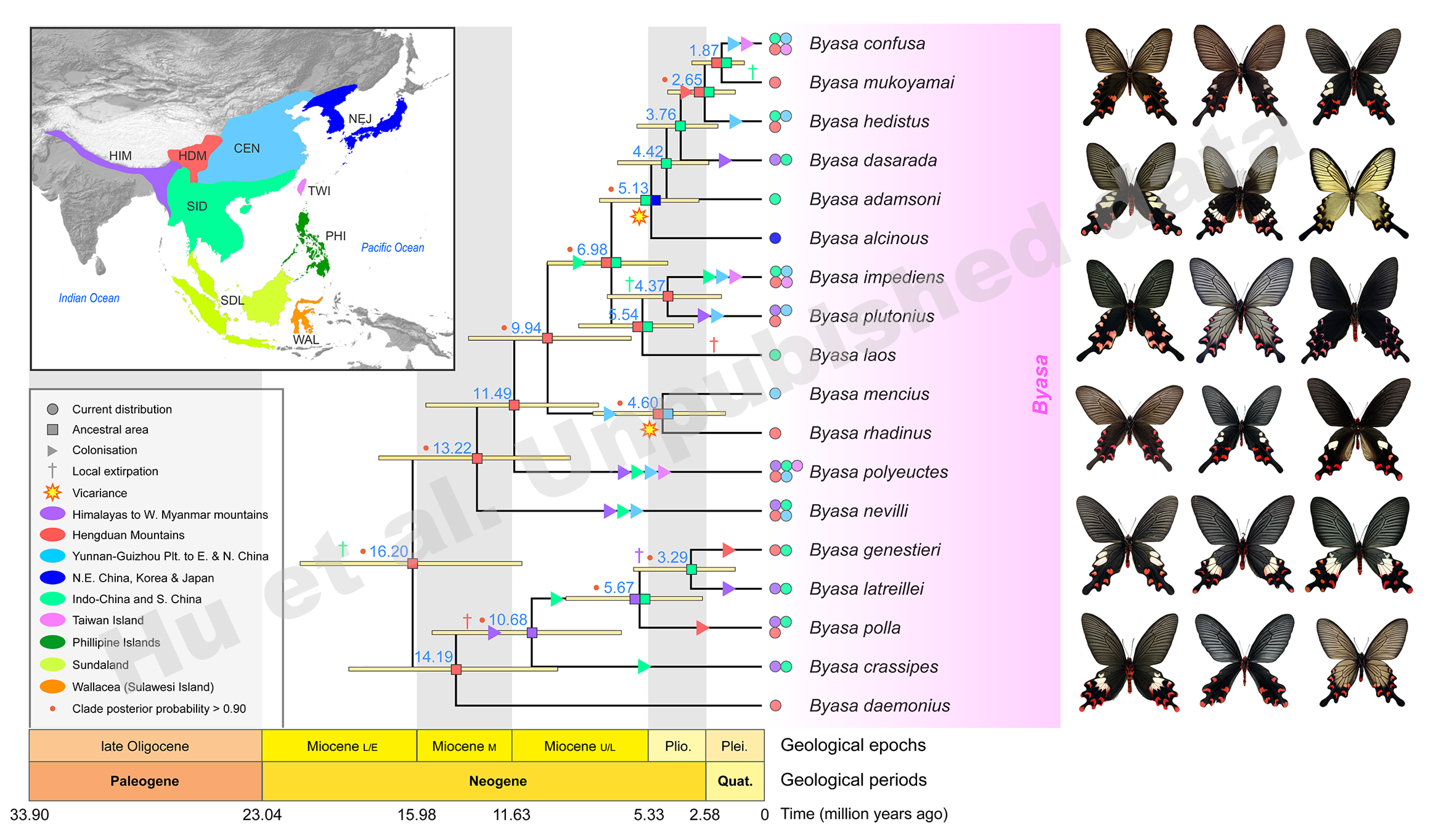

Supplement: Supplementary file 3 — Figure S3: Unpublished Bayesian dated tree and biogeographic history of Byasa. Node values represent median divergence times with coloured bars representing the 95% HPD. Squares on each node represent the estimated ancestral areas, while circles at the end of each branch represent current species distribution areas. Along branches, triangles indicate dispersal from ancestral areas to descendant distribution areas, daggers indicate extirpation from ancestral areas, and the colours of all these symbols mirror those on the biogeographic map in the top‐left corner. Explosion symbols represent vicariance events. [file ECE3-15-e72369-s002.tif]
